# Imatinib induces up-regulation of *NM23*, a metastasis suppressor gene, in human Hepatocarcinoma (HepG2) Cell Line

**Published:** 2017

**Authors:** Behta Keshavarz-Pakseresht, Seyed Ataollah Sadat Shandiz, Fahimeh Baghbani-arani

**Affiliations:** 1 * Department of Genetics and Biotechnology, School of Biological Science, Varamin-Pishva Branch, Islamic Azad University, Varamin, Iran*; 2 *Young Researchers and Elite Club, East Tehran Branch, Islamic Azad University, Tehran, Iran *

**Keywords:** Imatinib mesylate, *NM23*, metastasis, HepG2

## Abstract

**Aim:**

The present study investigated the anti-tumor activity of Imatinib mesylate through modulation of *NM23* gene expression in human hepatocellular carcinoma (HepG2) cell line.

**Background:**

Hepatocellular carcinoma (HCC) is considered to be the third leading cause of cancer related death worldwide. Down regulation of *NM23*, a metastasis suppressor gene, has been associated with several types of malignant cancer. Recently, effects of Imatinib mesylate, a first member of tyrosine kinases inhibitors, were indicated in research and treatment of different malignant tumors.

**Methods:**

Cell viability was quantitated by MTT assay after HepG2 cells exposure to Imatinib mesylate at various concentrations of 0, 1.56, 3.125, 6.25, 12.5, 25,50μM for 24 hours. Also, quantitative real time PCR technique was applied for the detection of *NM23* gene expression in HepG2 cell line.

**Results:**

There was a dose dependent increase in the cytotoxicity effect of imatinib. The real time PCR results demonstrated that inhibitory effect of Imatinib mesylate on viability via up regulation of *NM23* gene expression compared to *GAPDH* gene (internal control gene) in cancer cells.

**Conclusion:**

According to our findings, imatinib can modulate metastasis by enhancing *Nm23* gene expression in human hepatocellular carcinoma (HepG2) cell line.

## Introduction

Hepatocellular carcinoma (HCC) is one of the most common cancers; where over 600000 deaths occur annually worldwide (1). Chemotherapy, radiation and surgical therapy have been used for the treatment of HCC. Although conventional chemotherapy remained the only therapeutic approach after surgery, its use has been toxic (2). Therefore, novel therapeutic strategies are urgently needed for more profitable treatment. Knowing about the gene expression alteration throughout tumor progression has been the subject of intense method for targeting metastatic cascade and inhibition of tumor progression. Currently, metastasis suppressor genes (MSGs) are found to be significant in the regulation of cell invasion and metastasis cascades (3). Non-metastatic protein (Nm23) is a nucleoside diphosphate kinase that is an exhibited interesting attribute with regard to metastasis. Several studies have confirmed that the reduced *NM23* mRNA levels are related to different types of cancer cells with high metastatic potential (4, 5). 

Recently, Molecular targeted therapy is designed to inhibit important signaling pathways included in metastasis and apoptosis. Different preclinical and clinical studies on molecular targeted therapies have demonstrated that it has great promise in the treatment of various malignant tumors (6). Tyrosine kinases Inhibitors (TKIs) are promising anticancer agents that often induce apoptosis and slow progression growth of bone metastases in tumor cells (7). Imatinib mesylate ( ST-571, Gleevec; Novartis Pharma), is the first member of new class of TKIs that acts by inhibiting specific tyrosine kinases like Bcr-Abl fusion oncoprotein in chronic myeloid leukaemia (CML)(8), inhibits the activation of platelet-derived growth factor PDGF() (9),c-Kit and is currently under evaluation in research and clinical trials for several solid tumors (10,11). Also, it was shown that imatinib can decrease the progressive growth and migratory properties of prostate and colorectal cancers and promote apoptosis in a number of cell lines such as glioblastoma, retinal ganglion, gastrointestinal stromal tumors and leukemic cells (12). The aim of the current study was to investigate the effect of Imatinib mesylate on cell viability and anti-cancer effect through modulation of *NM23* gene expression in HepG2 human cell line. 

## Materials and Methods

***Cell culture***

The human hepatocellular carcinoma cell line HepG2 was obtained from the Pasteur Institute of Iran, Tehran. The cell line was grown in the Dulbecco’s Modified Eagle’s Medium (DMEM) medium supplemented with 10% fetal bovine serum (FBS),μ in a 5% CO_2_?

***MTT assay***

The degree of viability activity of Imatinib mesylate against HepG2 cancer cells was evaluated by 3-(4, 5-dimetheylthiazol)-2, 5-diphenyl tetrazolium bromide (MTT), assay. The grown cells at a density of 10,000 cells per well were seeded into 96-well microtiter plate and treated with different concentrations of Imatinib ranging from 0, 1.56, 3.125, 6.25, 12.5, 25 and 50μM?μ×100. The concentration that inhibits the activity by 50% (IC_50_) values of drug on HepG2 cells was evaluated using Graph-pad In Stat software.

***Total RNA extraction and cDNA Synthesis***

The HepG2 cell line was seeded into 6 well plates (5×10^4 ^cells/well) and incubated for 24 hours. Then, cells were treated with Imatinib mesylate (within IC_50_ dose) for 24 hours. According to the manufacturer’s instructions, total RNA was extracted using an RNA-isolation kit (Qiagen, RNeasy Plus Mini Kit 50). Complementary DNA synthesis was performed using commercially available kits (1st Synthesis Kit Roch, Germany) in a reaction mixture containing oligo dT Primer (50 μM), reaction Buffer 10x, MgCl_2_ (25mM), dNTP mix (1mM), total RNA (1μg), AMV reverse transcriptase (20 units), RNase inhibitor (40 units) and nuclease-free water. The amplification step for the reverse transcription was measured by ABI 7700 Thermal Cycler (Applied Biosystems Company, California, USA) as follows: 30 °C for 10 minutes, 42 °C for 50 min and 95 ºC for 5 min followed by cooling on ice for 5 min.

***Quantitative analysis of gene expression by Real-time-PCR***

μTable 1. The specificity of desired primers was checked by utilizing the BLAST program (www.ncbi.nlm.nih.gov/blast).

 The gene expression data was measured by using the comparative threshold cycle (Ct). the ΔCt value of *NM23* gene was obtained by subtracting the Ct of target gene from reference gene Then, to obtain ΔΔCt value, the ΔΔΔΔCt = [mCt NM23 – mCt GAPDH] _(treated sample)_ - [mCt NM23 – mCt GAPDH]_(untreated sample)_. Finally, the fold change in *NM23 *gene expression was calculated using the following formula: (Ratio formula= 2 ^-ΔΔCt^). The specificity of primer for amplification reactions was confirmed by melting curve analysis. Then, each real time PCR products were transferred into the well installed in 1.5% agarose gel in TBE and the fragments were visualized by ethidium bromide fluorescence method through trans-laminator system (UV dock, England).

***Statistical Analysis***

The statistical analyzes was calculated using SPSS statistical software version 22. The *P*-value of <0.05 data was considered statistically significant and was assessed using Students *t*-test. All experimental data were done in triplicate and presented as the mean ± standard error.

## Results

***MTT assay results***

**Figure 1:**
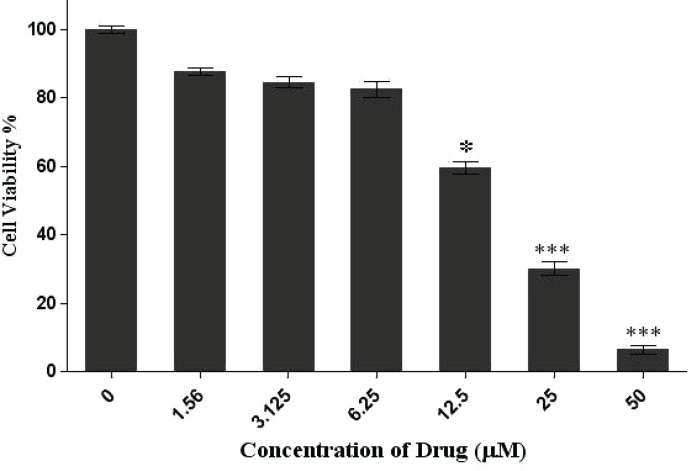
Cell viability assay of HepG2 cells after treatment with different concentrations of Imatinib within 24 h. The data was expressed as the mean±SD from 3 independent experiments. Results were statistically analyzed by a Student s *t*-test (*P 0.05; **P 0.01; ***P 0.001

*p* *p* *p* *p*(Figure1)._50_

**Table 1:** Characteristics of the Primers of *NM23* and *GAPDH* genes used in the real time PCR assay

**Gene**	**Primer sequence**
*NM23*	Forward: 5'- ATGGCCAACTGTGAGCGTACC -3 Revers: 5'- CATGTATTTCACCAGGCCGGC -3'
*GAPDH*	Forward: 5'-CGTCTGCCCTATCAACTTTCG-3' Revers: 5'-CGTTTCTCAGGCTCCCTCT-3'

**Figure. 2:**
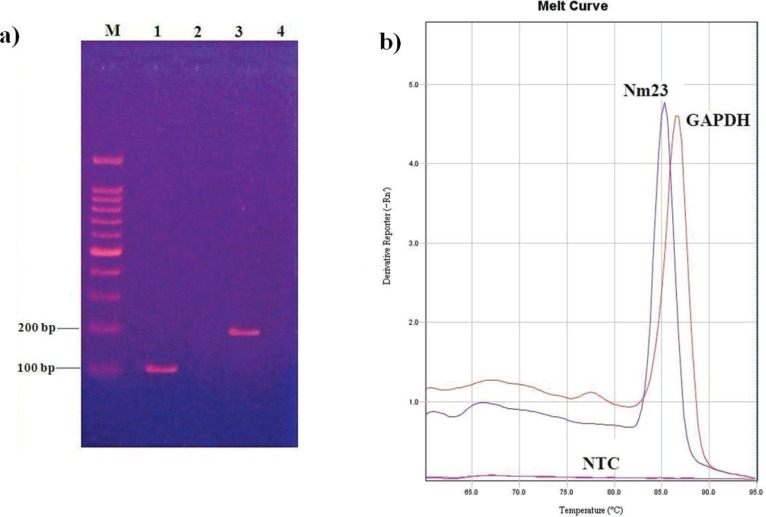
Gel electrophoresis and Melting curve analysis. (a) Gel electrophoresis of the PCR products. M: Molecular Size marker -100bp ladder. Lane1: 102 bp PCR product of *GAPDH *gene. Lane 2: Non Template Control for *GAPDH* gene. Lane 3: 204 bp PCR product of *NM23*. Lane 4: Non Template Control for *NM23* gene. (b) The melting curve at 86.5  C for *GAPDH* gene and 85.2  C for *NM23 *gene indicated the specific products that melt at the different temperature. Flat peak demonstrates Non Template Control: NTC

**Figure 3:**
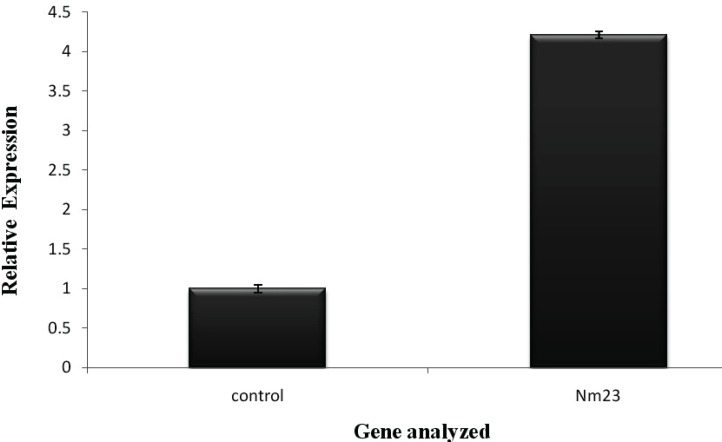
The impact of Imatinib mesylate to expression of NM23 mRNA levels in 21.89 M of drug concentration toward HepG2 cells after 24hours. The expression of mRNAs was analyzed by Real-time PCR and normalized by *GAPDH* expression. P-value of <0.05 versus control group (one-way ANOVA analysis followed by the Student’s t-test

***Melting curve analysis***

For quantitative gene expression analysis by Real-time-PCR, the melting curve plot was carried out based on dF/dT derivation (y axis) and the temperature (x axis). Our observation revealed a single product formation and screen for primer dimmers for each gene. The melting peaks have been drawn at 85.2°C for *NM23* gene and 86.5°C GAPDH gene as shown in Figure 2a. Moreover, gel electrophoresis results revealed the specific amplification of genes according to their sizes (Figure 2B).

***Relative quantification analysis using amplification plots***

The relative gene expression between untreated and treated samples can be measured by the difference in their Ct values of exponential phase. The mCt, mCt and Ct value for NM23 and GAPDH genes were evaluated in IC_50 _concentration of imatinib mesylate. The NM23/GAPDH gene expression ratio equals to 2 ^-^ ^Ct^. The expression of NM23 mRNA level was±μFigure.3). 

## Discussion

Disturbance in the cell signaling pathways clearly has been studied in advanced HCC due to the resistance to therapy and progression of disease that causes the treatment choice limitation (13). Consequently, Tyrosine kinases Inhibitors are promising anti-cancer agents which often interfere with a specific molecular target in cell controlling signaling pathways in neoplastic cells (14, 15). These targets include modulators of apoptosis, cell-cycle proteins, growth factor receptors, and molecules involved in angiogenesis and invasion, which are important for homeostasis and development in normal tissues. Many studies have been reported the effect of Imatinib mesylate on several human solid tumors, including small cell lung cancer (16), thyroid cancer (14), and ovarian (17).

The use of Imatinib in the molecular targeting of cell signaling genes and proteins in HCC cells has not completely investigated to this point targeting the *NM23* gene. In the current study, we demonstrated that Imatinib exerted a dose-dependent inhibitory effect on the viability of human hepatocellular carcinoma HepG2 cell line. Treatment of HepG2 with Imatinib induced the morphological changes that verify the expansion in apoptotic cell population. Most cancer mortalities are induced by the progression of tumor metastasis; hence the most important contributor to cancer related morbidity and mortality (18, 19). 

Identification of the gene expression during tumor progression has been the great significant of intense method for prognosis and therapy. The amount of *NM23* gene expression has a significant role in targeting tumor metastasis. Loss of *NM23* expression has been correlated with the degree of metastasis and undesirable clinical prognosis in various types of human carcinoma (18). Also, it has been demonstrated that the NM23 protein is participated in the regulation of several cellular responses such as differentiation, endocytosis, development, and apoptosis (20,21). 

Several researchers claim that there is an important relation between reduced expression of *NM23* and the occurrence of metastasis in breast cancer, malignant melanoma, gastric cancer and hepatocellular cancers while there is completely opposite in case of adenocarcinoma of the lung, neuroblastoma. In 1995, Lin, *et al.* reported correlation between high *in vitro* invasive capacity and a low NM23 protein level when analyzing eight human liver cancer cells (22). Fujimoto, *et al.* (1998) observed that the nm23 protein level was obviously decreased in poorly differentiated HCC cell lines, HuH-1and HuH-2 and the hepatocarcinoma cell line, HepG-2 (23). In 1994 yamaguchi *et al* reported that NM23 expression was conversely related to metastasis potential of HCC (24). Recently, it has been reported that the alteration of P53 tumor suppressor gene and down regulation of *NM23* gene are both more prone to metastasis (21). The interaction between P53 and Nm23 may lead to cell cycle arrest and apoptosis. It has been demonstrated the NM23 protein with STRAP (serine threonine kinase receptor associated protein) promotes the association between p53 and NM23, resulting in the apoptosis and cell cycle arrest (21). The consequential finding in our study was indicating that Imatinib can up-regulate *NM23 *gene expression in human hepatocellular carcinoma HepG2 cell line which has not yet been studied. To the best of our knowledge, increased expression of *NM23 *was changed significantly 4.20 fold at IC_50_ of Imatinib concentration respect to control gene. Therefore, Imatinib mesylate remains a promising candidate for the treatment of hepatocellular carcinoma in the future. To evaluate if imatinib treatment results in metastasis induction, our results demonstrated that imatinib mesylate induces a metastasis mediated by up regulation of metastasis suppressor gene *NM23* gene expression on HepG2 cells. 

In this study, we demonstrated that treatment with imatinib for 24 h induces a dose-dependent inhibitory manner on the HepG2 cell line. Also, imatinib mesylate induces up regulation of *NM23 *expression, metastasis suppressor gene, in hepatocellular carcinoma HepG2 cells. Thus, for a definitive conclusion, imatinib may be a good candidate for utilization as an inhibitor of the growth of other cancer cell lines as well as *in vivo* animal tumor models.
